# Procedure for the selection and evaluation of prefabricated housing buildings for the implementation of green roofs in the context of Urban Heat Island mitigation. The example of Wrocław, Poland

**DOI:** 10.1371/journal.pone.0258641

**Published:** 2021-10-14

**Authors:** Justyna Rubaszek, Mariusz Szymanowski, Adam Michalski, Radosław Tatko, Marta Weber-Siwirska

**Affiliations:** 1 Department of Landscape Architecture, The Faculty of Environmental Engineering and Geodesy, Wroclaw University of Environmental and Life Sciences, Wrocław, Poland; 2 Institute of Geography and Regional Development, Faculty of Earth Sciences and Environmental Management, University of Wrocław, Wrocław, Poland; 3 Institute of Geodesy and Geoinformatics, The Faculty of Environmental Engineering and Geodesy, Wroclaw University of Environmental and Life Sciences, Wrocław, Poland; 4 Institute of Building Engineering, The Faculty of Environmental Engineering and Geodesy, Wroclaw University of Environmental and Life Sciences, Wrocław, Poland; Northeastern University (Shenyang China), CHINA

## Abstract

The assessment of the suitability of existing buildings for implementation of green roofs is an important research issue, especially in the context of Urban Heat Island (UHI), the negative impacts of which are locally exacerbated by the global warming. The studies carried out so far have covered a variety of buildings and have taken into account a range of different conditions. Relatively little attention has been paid to the possibilities of greening the roofs of prefabricated apartment blocks from the second half of the 20th century in the context of the potential climate effect. Yet, these buildings are found in many cities around the world, and seem in fact attractive for greening. In view of the above, we proposed a three-stage investigatory procedure to: (I) identify and classify buildings based on the number of floors and the rooftop available area; (II) select buildings by designating priority areas depending on the highest UHI intensity and roof density; (III) analyse the roof load capacity to develop retrofit scenarios. The procedure was applied to prefabricated housing estates built in the 1970s and 1980s in Wrocław, Poland. The research shows that there are 1962 buildings of different heights and roof area of 722405 m^2^, of which 480 buildings with a roof area of 122749.1 m^2^ were selected for greening within priority areas. The structure of the studied roofs was not designed to carry additional loads, which requires the application of complementary solutions. Scenario 1 assumes extensive greening provided that the existing ventilated roof is strengthened, scenario 2 –semi-intensive greening, which however requires the conversion of the ventilated roof to a non-ventilated one. The presented procedure can be applied in any other city with prefabricated apartment blocks and available UHI data, and serve to support the decision to implement green roofs to mitigate UHI.

## Introduction

Planning and designing green roofs provide several environmental and social benefits and thus allows to achieve a more sustainable, resilient, healthy urban environment [1–6]. Many countries are making strong initiatives to apply the green roofs at the newly erected as well as already existing buildings [6]. Therefore, identifying the technical possibilities and limitations of greening the existing rooftops is an important research issue influencing decisions on future modernization investments.

One of the main motivators for implementing green roofing systems is minimizing the UHI effect [7]. The roofs of buildings occupy as much as 27.8% of all impermeable surfaces in urban areas [8] and are among the hottest city surfaces during the day [9]. Materials covering traditional roofs, such as concrete or asphalt absorb and store excessive amounts of heat, and thus adversely affect the urban thermal environment [10]. Covering the building with greenery, including replacing its roof with a green roof, is considered one of the ways of regulating the local climate, including the reduction of the UHI [10, 11].

Minimizing the impact of UHI is important for many reasons. Elevated temperatures cause higher energy consumption and raise the peak electricity demand [12–15], influence air pollution [16], reduce human thermal comfort and pose a threat to human health [17, 18]. It is also argued that UHI’s energy, environmental and social impacts are further exacerbated by global climate change [19, 20].

The desired cooling effect of green roofs results mainly from evapotranspiration and shading [11]. As shown by numerous studies to date, the effectiveness of green roofs depends mainly on the plant species used, including their size and foliage density [21–25]. Therefore, not every type of green roof will have the same efficiency in terms of lowering the temperature. For instance, a field experiment conducted by Zhang et al. [24] in Hangzhou, China, indicates that the use on a prefabricated green roof of *Sedum lineare* does not cool ambient environment but leads to an increase in surrounding air temperature on extremely hot days. Studies on two types of extensive greening carried out by Jim et al. [22] show that a sedum roof stores some heat which in turn raises the temperature inside and outside the building and intensifies the UHI. Thus, extensive roofs, which due to their low weight are generally recommended for use on existing buildings, will not always be effective in terms of temperature reduction.

In turn, the mesoscale impact of green roofs increases with the size of the green coverage. Founded on a single urban block, they create a small ’cool island’–installed on many roofs they provide more general UHI mitigation benefits [26]. Large-scale application of green roofs could reduce the ambient temperature from 0.3 to 3°C [10]. It is therefore legitimate to transform rooftops into green roofs on as many buildings as possible.

The effect of green roofs on the reduction of the ambient temperature is also dependent on urban morphology (e.g. urban pattern, the height and layout of buildings) [26–31]. For example, a scattered layout of buildings with green roofs proves to be better in terms of local temperature regulation compared to enclosing and array layouts [27]. The above research also proves that the influence of green roofs on the temperature at the pedestrian level largely depends on the height of the buildings on which they are installed–the lower the buildings, the more significant impact of green roofs [e.g. 28–31].

However, this temperature-lowering effect can be enhanced or weakened by atmospheric factors, e.g. green roofs in upwind zones are able to generate the most favorable cooling effect, while green roofs in downwind zones make slight differences to pedestrian thermal environments [31]. It is also known that within the green roof itself, the temperature will be different directly on the roof surface and at the height of a sitting or standing person [32]. The lowering of the temperature is also influenced by architectural elements such as pergolas and other shading structures [32], as well as the maintenance of the roof, including its irrigation [24, 33].

In studies on the adaptation of the city’s form in order to improve the thermal environment, various indicators are considered, e.g. building density, building height, floor area ratio, sky view factor [34–39]. When examining the possibility of greening the roofs of existing buildings, the indicators relating to the roofs are of particular importance. The most important ones are roof slope, rooftop available area for green roof applications, roof load capacity [see 40 for more]. The roof slope and rooftop available area can be analyzed for larger areas or entire cities based on freely available geospatial vector data, orthoimagery, and very high spatial resolution remote sensing data using the Geographic Information System (GIS) [e.g. 41–44], and finally–the load-bearing capacity of the roof, based on in situ studies and construction designs [45–49].

Previous research on the potential of existing roofs to greening have been carried out in the context of various environmental issues. In addition to the assessment of the possibility of greening, various benefits have been demonstrated, e.g. in terms of reducing rainwater runoff or energy consumption [41, 49, 50]. Part of the research was aimed at identifying areas (referred to as priority areas or zones) where the greening of roofs is justified by one or several environmental factors [40, 50–54].

For example, Velázques et al. [55] while determining the priority areas for the introduction of green roofs on existing buildings, takes into consideration vegetation deficiency, pollution intensity, traffic congestion and population density, Silva et al. [40] on the other hand focus on green surface area, high greenery, i.e. trees, whereas Zhou et al. [55] in their studies concentrated on rainwater runoff. Climatic criteria were taken into account in the works of Grunwald et al. [51], Langemeyer et al. [53]–the first authors took into account the parameters from the climate function map with climatopes, the second–the air temperature based on the annual measurements. However, none of the authors while specifying the priority areas referred directly to the UHI magnitude.

Assessments of the possibility of greening the existing roofs were made on the scale of entire cities [e.g. 40–44] or related to selected types of buildings [e.g. 45–49], but so far little attention was paid to prefabricated apartments blocks commonly erected in the second half of the 20th century [55–57]. The technology that was new at that time was seen as a ’modern’ response to the acute housing shortage, which occurred in many European countries after World War II. To this day, large-panel housing estates are inhabited by a significant percentage of the population–in Western Europe this percentage is typically below 10%, while in post-socialist countries this percentage is around 40% [58]. In Poland alone, the number of flats constructed in Soviet era buildings is 5,200,600, which is higher than in Lithuania, Ukraine, Estonia, and former East Germany [59].

Prefabricated apartment blocks mostly have flat roofs with large and uniform surfaces, designed according to the style of modernist housing architecture. In many cities destroyed by World War II, they were built not only on the outskirts, but also in city centres, where UHI is highest and elevated temperatures are particularly noticeable. Green roofs introduced on their rooftops could therefore have a positive effect on reducing temperatures [see 60, for more].

In the studies of prefabricated buildings, the focus was mainly on spatial, functional and social problems that have occurred in prefabricated housing estates [55, 61–65]. The issues of safety and strength of the structure as well as modernization were also discussed [66–69]. The latter, however, in most cases included various types of technical solutions, but without the use of green roofs, which is confirmed by, among others case studies collected under the COST UCE program(European COoperation in the field of Scientific and Technical research under the Urban Civil Engineering Technical Committee) [70–72].

Taking into account the above issues and trying to fill the gap identified in the previous research, in this study we decided to address the following questions:

1. how to select prefabricated apartments blocks to choose those where green roofs can have the greatest impact on UHI reductions?
2. whether and how can roofs of prefabricated apartment blocks be greened?

For this purpose, we proposed a procedure consisting in the identification and selection of buildings and the assessment of the possibility of greening their roofs as a way to mitigate the UHI effect. The study was carried out for the city of Wrocław–a typical example of a post-socialist, central European, medium-sized city with a clearly visible UHI and a large share of prefabricated housing buildings.

## Materials and methods

### Study area

**Fig 1:**
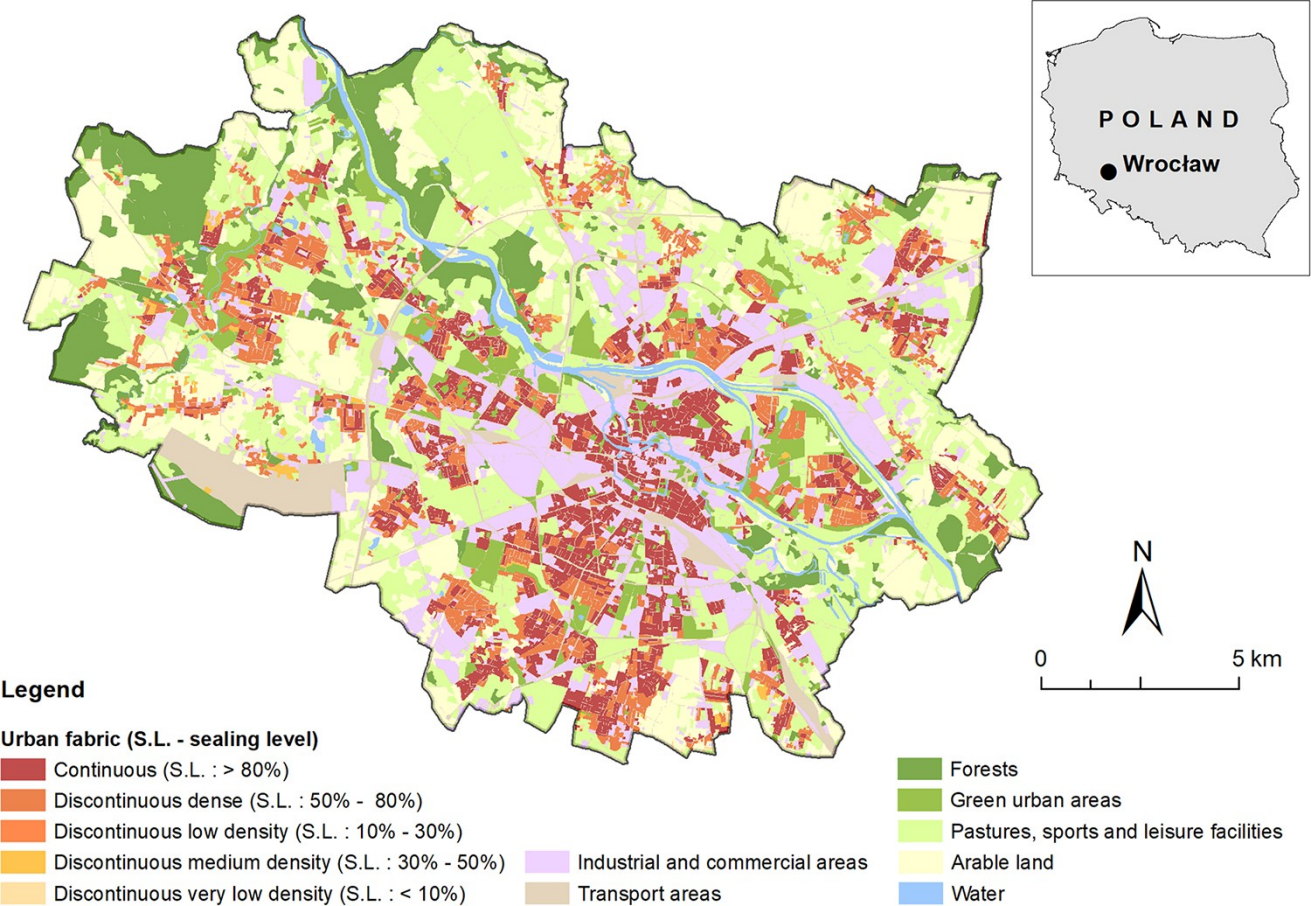
Land cover map of the city of Wroclaw (Poland) based on data from Urban Atlas 2018 (https://land.copernicus.eu/local/urban-atlas/urban-atlas-2018).

The study was performed in the city of Wrocław, located in SW Poland (51°N, 17°E). In terms of population, Wrocław is the fourth largest city in Poland (642869 inhabitants) and fifth in terms of surface area (292.82 km^2^) [73]. Approximately 31.4% of the city area comprises built-up land, 28.9% agricultural areas (cultivated and bare fields), 36.6% urban green space with semi-natural forests and grasslands and 3.1% water (Fig 1).

Wrocław is located in a temperate, transitional (maritime continental) climate zone, with the mean annual temperature of 8.8°C. The UHI phenomenon in Wroclaw has been investigated and described on the basis of data for the period April 1997–March 2000 including detailed average, extreme, and frequency of UHI values. These data show that the increase in mean annual temperature in the city centre and the inner city is 1.0K. The maximum temperature difference between the city centre and suburban areas can exceed 9.0 K, and the average UHI at night is two to three times higher than the average value during the day, which is typical for cities of similar size to Wrocław. Positive UHI values in the central parts of the city are observed in> 96% of the night hours and> 80% during the day, with a strong UHI effect (> 5.0 K) measured in 3.8% of the night hours and only randomly during the day. The annual cycle of UHI intensity depends on meteorological conditions and artificial heat release, so the most optimal conditions for UHI occur in the warm season, with the highest values recorded in Wroclaw in May and August due to increasing convective cloudiness in mid-summer (June, July). A secondary maximum of the UHI magnitude was observed in January, with lows in October and February [74, 75].

**Fig 2:**
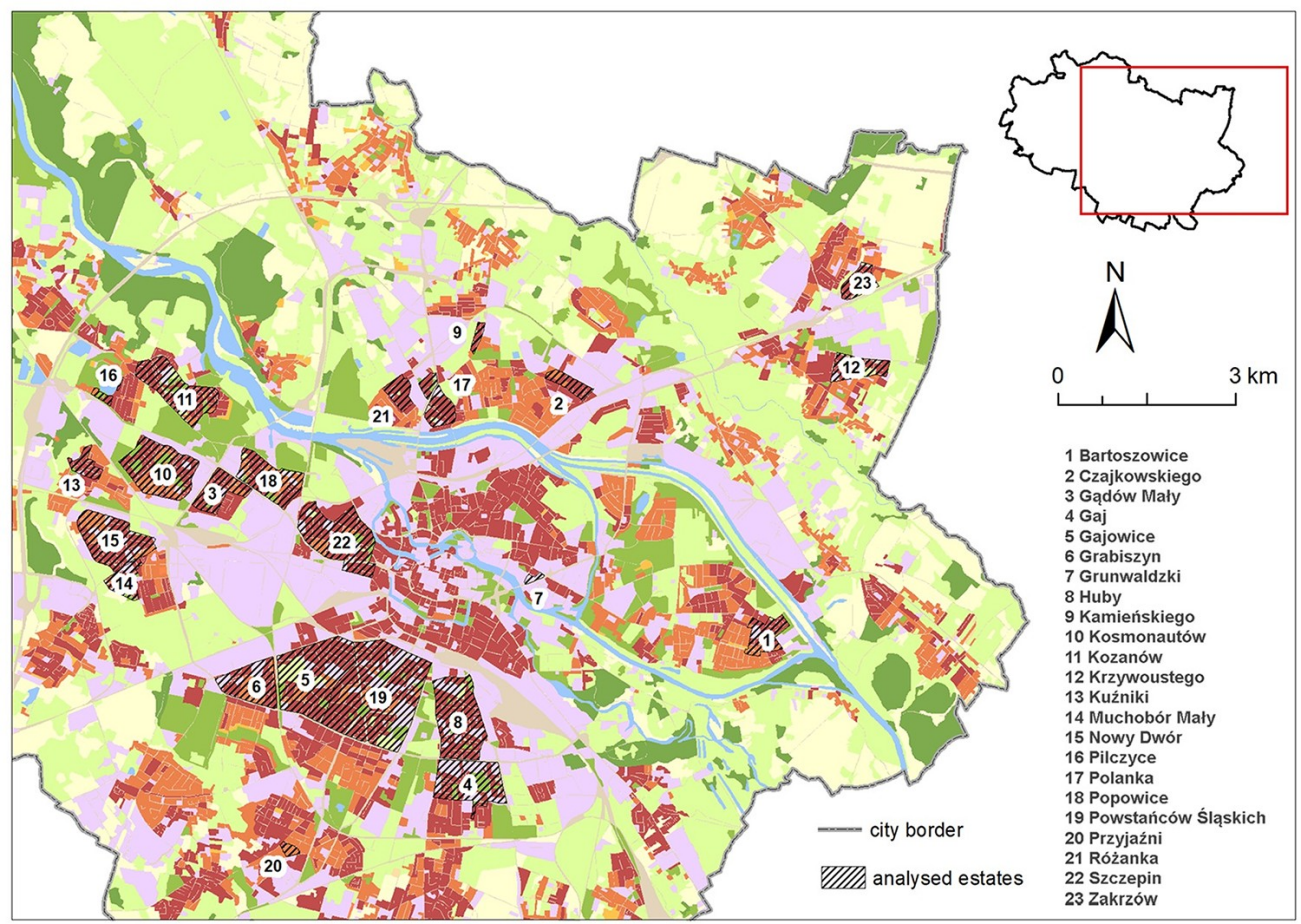
The surveyed housing estates in Wrocław with prefabricated apartment blocks built in the years 1970–85 presented on the land cover map of the city of Wroclaw (https://land.copernicus.eu/local/urban-atlas/urban-atlas-2018).

The research presented in this article is based on prefabricated apartment blocks located in Wrocław housing estates constructed in the period 1970–1985. 23 sites in total were included in the study (Fig 2).

**Fig 3:**
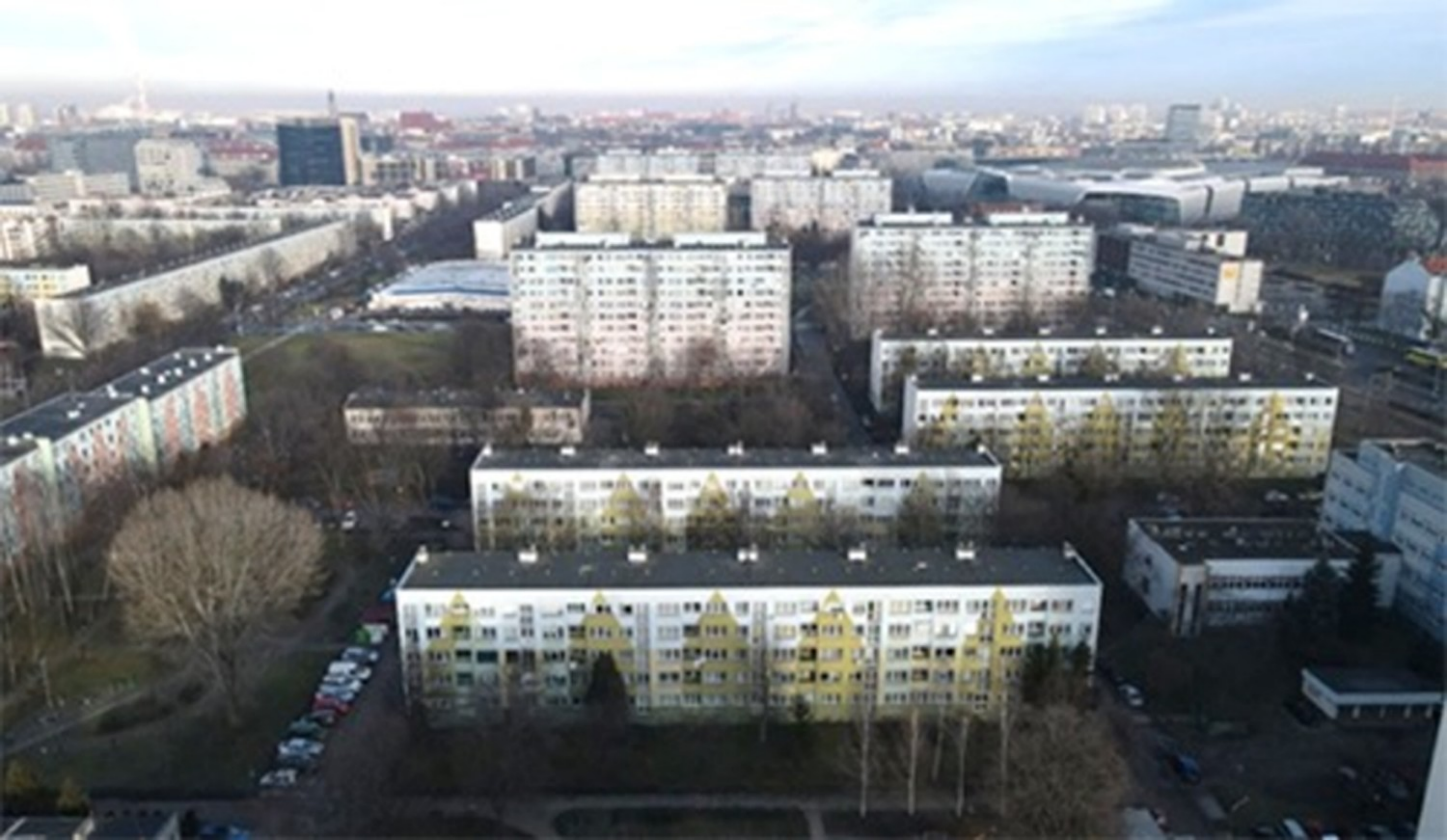
Example of prefabricated housing in Wroclaw–a fragment of Powstańców Śląskich housing estate.

Some of the estates were established in previously unbuilt areas, others were incorporated into the historical structure of the city to fill the empty spaces where buildings had been demolished as a result of the warfare in 1945: Gajowice (5), Grabiszyn (6), Huby (8), partly Bartoszowice (1), Powstańców Śląskich (19, Fig 3), Grunwaldzki (7). The other of the surveyed districts were built on undeveloped areas, with a previously undeveloped urban fabric. These include: Czajkowskiego (2), Gądów Mały (3), Gaj (4), Grunwaldzki (7), Kamieńskiego (9), Kosmonautów (10), Kozanów (11), Krzywoustego (12), Kuźniki (13), Muchobór Mały (14), Nowy Dwór (15), Pilczyce (16), Polanka (17), Popowice (18), Przyjaźni (20), Różanka (21), Szczepin (22), Zakrzów (23). Delimitation of the estates for the purposes of the study was made on the basis of the literature [76, 77] and the analysis of the distribution of prefabricated housing estates. The boundaries of the estates are not the same as the boundaries of the administrative units, also called estates, into which the city of Wroclaw is divided, but they cover the areas planned and developed as coherent housing units in the years 1970–85.

### Data

The research was carried out on the basis of:

■ field inspections supported by Google Street View (GSV)
■ publicly accessible database of topographic objects BDOT10k (www.geoportal.gov.pl) updated for 2018
■ publicly accessible data on the height of buildings (https://geoportal.wroclaw.pl/en/resources/) updated for 2015
■ publicly accessible orthophotomap 2020 made on the basis of digital images acquired in the 2nd– 3rd quarter of 2020, with field resolution of 25 cm/pixel (https://geoportal.wroclaw.pl/en/resources/)
■ UHI normalized map from 2001–2002
■ construction designs of buildings obtained from housing cooperatives.

For the purpose of the study, an averaged map of the UHI in Wrocław was prepared. The last detailed measurements of air temperature with the use of mobile stations, which allowed for in-depth studies on the spatial structure of the UHI in Wroclaw, were taken in the years 2001–2002. Seven comprehensive measurement sessions were then performed in different, yet conducive to the formation of the UHI of several degrees, weather conditions, which made it possible to elaborate the first detailed maps of the UHI in Wroclaw [75]. A decade later, the application of methods dedicated to the conditions of spatial non-stationarity (to which the distribution of air temperature in Wrocław in the analysed cases was subjected) allowed for the improvement of the quality of these maps [78]. Since the UHI magnitude is subject to strong spatial variations in the diurnal and annual cycle, the elaboration of the mean UHI map encounters obvious difficulties. For the purpose of this work, a procedure for processing UHI maps was implemented. Each map was normalised to a range of 0–1, and then the values from the seven maps were averaged to obtain a normalised UHI map–nUHI (normalised UHI). Since the mean annual UHI in the central areas of the city is about 1 K (see also section Study area), the map obtained can be regarded as an approximation of the mean annual UHI magnitude. Obviously, over the nearly 20 years since the measurements were made, a number of land cover changes have been observed in Wrocław. Nonetheless, it should be emphasised that these changes were dispersed and covered in the period 2006–2018 (Urban Atlas– https://land.copernicus.eu/local/urban-atlas) relatively small areas (6.5% of the city area), mostly located outside the city centre. Within the area of the analysed neighbourhoods, land cover changes covered 2.8% of the land area and mainly involved the introduction of new development elements or a change in the class of development (increase in density). Although the indicated land use–land cover (LULC) changes certainly led to changes in the UHI range, given their spatially dispersed distribution, it may be assumed that these were local rather than mesoscale alterations.

## Methods

**Fig 4:**
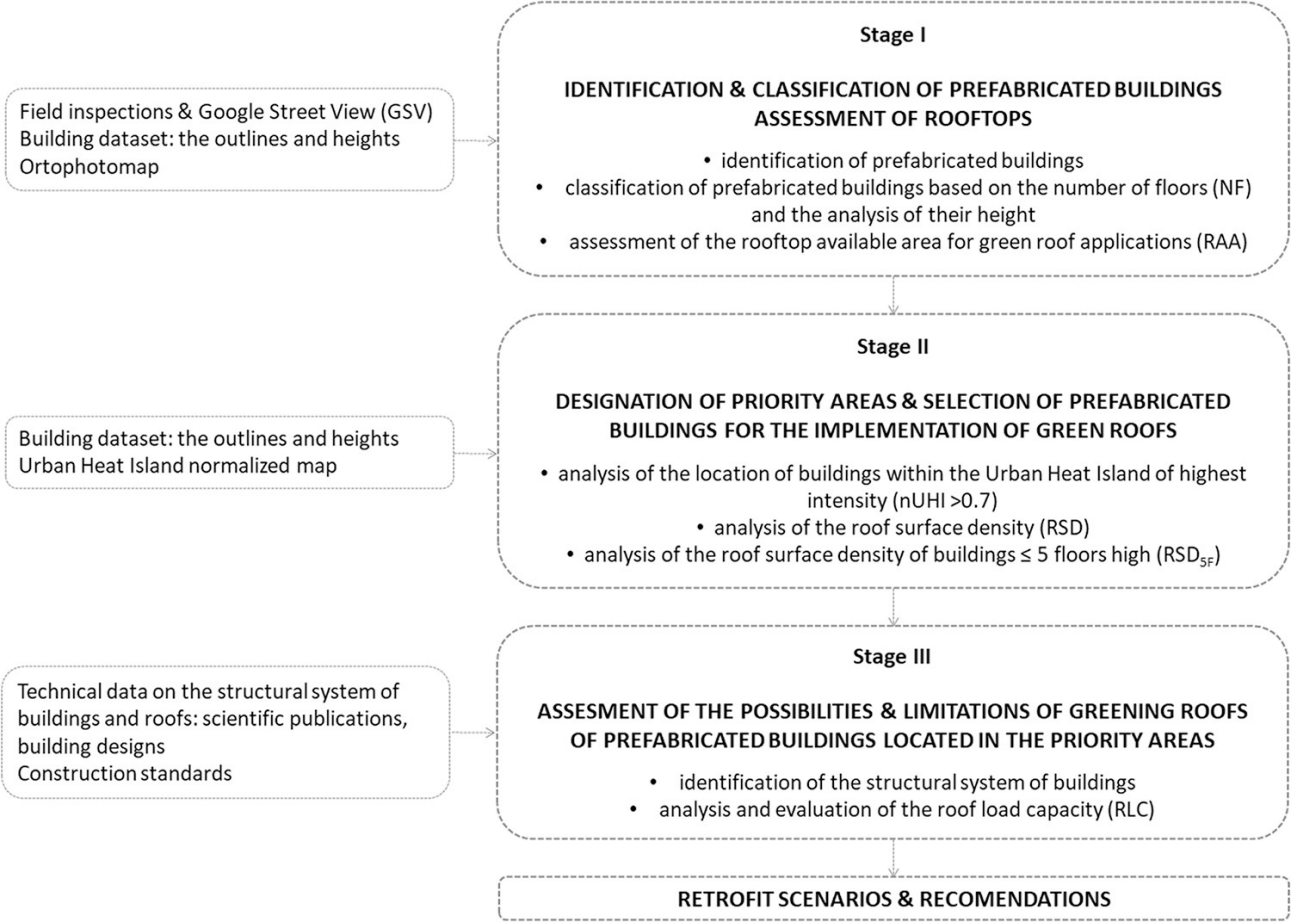
Flowchart of the study.

The flowchart of this study is as follows (Fig 4).

The research was conducted in three stages. Initially (Stage I), all prefabricated residential buildings located within the boundaries of designated housing estates were identified; buildings were classified according to the number of floors (NF) into two groups: those having up to 5 floors and the ones with more than 5 floors. This classification resulted from the types of prefabricated buildings built in Wrocław; they were mainly erected as 5-floors and 11-floors apartment blocks [77, 79].

Identification of buildings in individual housing estates was made on the basis of field inspections and supported by google street view analysis. The use of Google Street View (GSV) in surveys in urban areas is very helpful as it facilitates and accelerates the time-consuming field investigations [cf. 80, 81]. Building outlines were obtained from the publicly available BDOT 10k topographic objects database. It was assumed in the survey that the outline of the building at the ground level is equal to the roof surface–roofs of the surveyed buildings are limited by gable walls, and if they have eaves, it is usually on one side, 20 to 30 cm wide. This was considered to be a minor deviation not relevant to the housing estate and city scale result. The height of the buildings was checked on the basis of publicly accessible data (point ESRI Shapefile dataset containing building centroids with maximum height attribute). All data was processed using ArcGIS.

Then, an analysis of the rooftop available area for green roof applications was carried out. The rooftop available area for green roof applications (RAA), next to roof slope (RS), what has already been mentioned, is one of the most important parameters used in the external assessment of roofs in terms of their greening potential [40–42, 48, 49, 51, 82]. The RAA can be limited by the presence of various types of structural elements, such as chimneys, skylights, lift shafts or air-conditioning installations, which excessively divide the roof area, making it difficult to establish green roofs. Roofs with homogeneous undivided surfaces are considered the best roofs for greening [83]. The roofs of the studied buildings originally contained only chimneys and lift shafts, but they did not divide the roof surface in such a way that the implementation of a green roof would have been impossible. However, since their construction period, the building roofs themselves may have been altered and various types of new elements and installations may have occupied the roof surface. It was assumed that roofs with approximately ½ of their surface occupied by new elements, e.g., skylights, superstructures, air-conditioning installations, had limited potential for the installation of green roofs for implementation reasons; with approximately ¾ of their surface occupied, it was considered that these roofs were not suitable for greening. With regard to photovoltaics, which are becoming increasingly common on roofs, as moveable objects they are not considered to be a limiting element for greening the roof [41, 42], the more so as combining both technologies is not only possible but also beneficial [84].

The RAA was estimated on the basis of building outlines and the orthophotomap from 2020 using ArcGIS.

Subsequently (Stage II), priority areas were designated where the establishment of green roofs is particularly important due to the possible local reduction of UHI.

The following indicators were adopted for selecting priority areas:

- location within the UHI of highest intensity
- large roofs surface density
- large roofs surface density of buildings with up to 5 floors.

The threshold for the location within the UHI of highest intensity was adopted as nUHI>0.7. This threshold may be changed depending on the distribution of nUHI in the city and the location of the surveyed buildings in relation to nUHI.

The location of buildings within the range of areas of large roofs surface density and large roofs surface density of buildings with up to 5 floors provides the opportunity to achieve a UHI change effect beyond the microscale impact. If the green roof area introduced was relatively large over a small area, its impact could be noticeable over a range of hundreds of metres. Such a mesoscale effect could lead to a permanent reduction in the intensity and change in the spatial structure of the UHI [10, 26]. In addition, a large roofs surface density of buildings with up to 5 floors (RSD_5F_), i.e., the assumed location of green roofs relatively low, allows for a better mitigation effect.

If the biologically active surface is high, heat exchange fluxes are directed to the urban boundary layer and not to the urban canopy layer and do not directly affect the UHI (in the classical sense, at the height of meteorological measurements). Lower-lying green surfaces provide an opportunity for local changes in heat balance within the urban canopy layer [23, 26–31, 85].

In order to apply the large roofs surface density (RSD) and roofs surface density of buildings with up to 5 floors (RSD_5F_) indicators, the density of the roofs area was calculated using the Point Density tool in ArcGIS, which calculates a magnitude-per-unit area from point features that fall within a neighbourhood around each cell. In these calculations the building is represented by its centroid, to which the roof area attribute is assigned. Since the typical source area of a meteorological signal in built-up areas seldom exceeds 1 km [86], but is stronger in closer proximity, the reference area (neighbourhood) was defined as a circle with a radius of 500 m. As a result, maps of roofs area density per unit land area were obtained. The sites with the highest proportion of roofs area were then determined by reclassifying the density maps. An arbitrary decision was taken that the threshold value would be a density equal to the average + 3 standard deviations. The superimposition of the reclassified maps made it possible to determine priority areas where all three criteria are simultaneously met.

In the final phase of the study (Stage III) a roof load capacity analysis of buildings located in priority areas was performed. The RLC is considered as one of the indicators that, together with RAA and RS, determines the possibility of introducing a green roof [45–49]. While the RAA and RS can be determined by an external analysis of the building, knowledge of the roof structure is necessary to assess the RLC.

For the buildings under study, the RLC analysis was performed based on:

- the technical data contained in the literature on the structural system in which the buildings were constructed [79, 87]
- technical documentation for a typical prefabricated building
- the snow load standard [88] valid in the period of building erection and the current snow load standard adapted to the European norms [89].

The research was aimed at verifying the load for which the roof of buildings located in priority areas was designed, what load reserve existed, whether and by how much this reserve was exceeded when the layers of selected green roof types were loaded. In this way, technologically possible scenarios for the reconstruction of building roofs were determined.

The selection of green roof types was based on weight, followed by the possibility to introduce the most diverse vegetation possible, due to potential microclimatic, natural, and aesthetic benefits. The issue of maintenance of the green roof was also taken into account, with the selection of roofs that do not require regular care.

## Results

**Fig 5:**
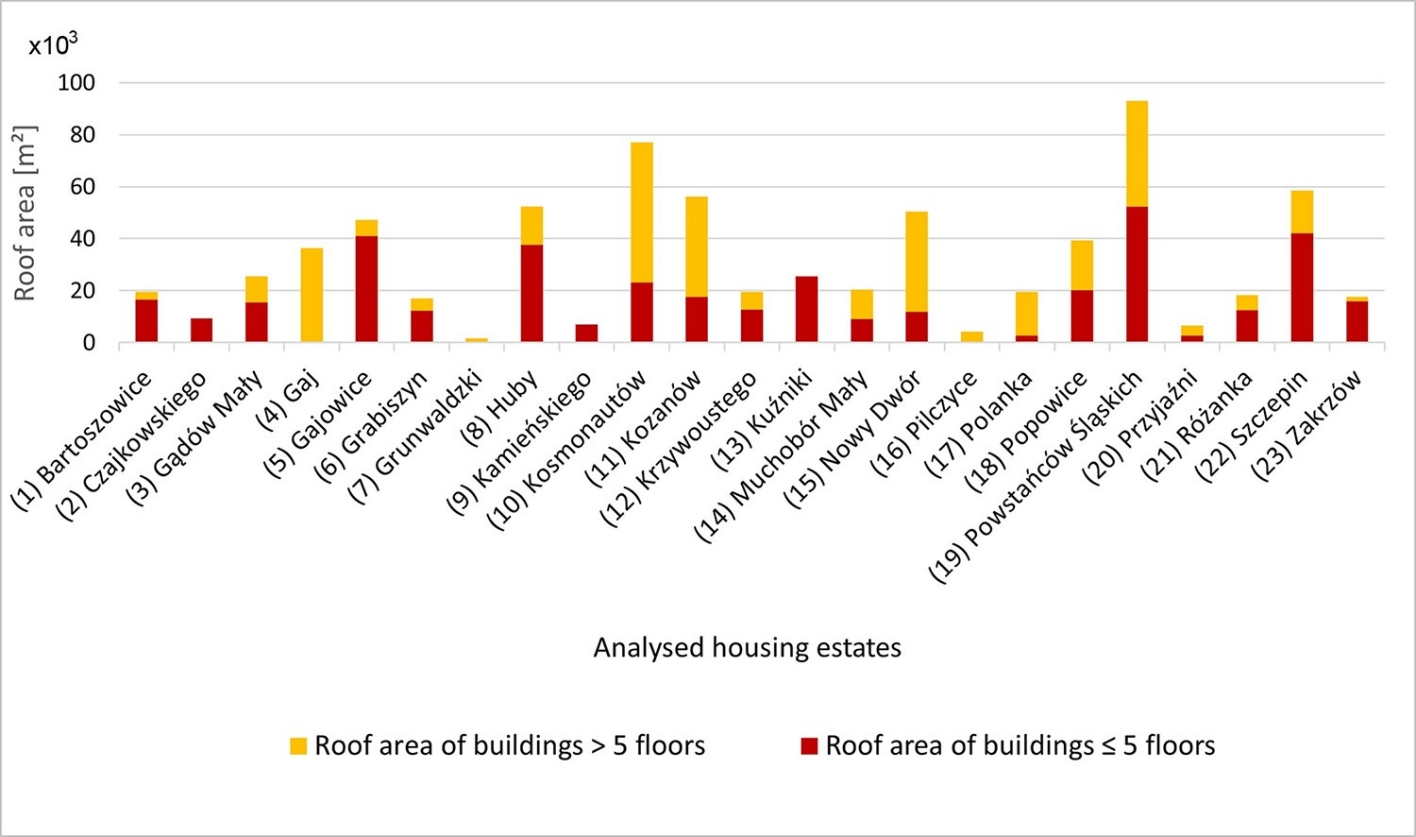
Roof area [m^2^] of prefabricated buildings distinguishing between buildings up to 5 floors and over 5 floors in the analyzed housing estates. Numbering of estates in accordance with Fig 2.

The investigations of the first stage showed that there are 1962 prefabricated apartment blocks in Wrocław housing estates, which accounts for 2.57% of all buildings in the city (in total there are 65,523 buildings in Wrocław). The total area of roofs in the investigated buildings is 722,404 m^2^, which represents 0.24% of Wrocław’s area. Buildings with up to 5 floors are up to 17.5 m, with most of the buildings below this height. In the group of buildings with more than 5 floors, 11-storey buildings predominate. They are up to 38 m high. The total number of buildings of up to 5 floors amounts to 1,318 (67.2%), and above 5 floors– 644 (32.8%). The total roof area of buildings with up to 5 floors is 388,147 m^2^ (53.7%) and that of buildings having more than 5 floors is 334,258 m^2^ (46.3%). The largest area of roofs of buildings with up to 5 floors is found in the districts of Powstańców Śląskich (19), Szczepin (22), Gajowice (5), and Huby (8), while the area of roofs of buildings having more than 5 floors is found in Kosmonautów (10), Powstańców Śląskich (19), Nowy Dwór (15), Kozanów (11), and Gaj (4) (Fig 5).

The RAA analysis reveals that the roofs of the buildings have not been re-structured and there are no new building elements that prevent the installation of a green roof; instead, photovoltaic cells have been introduced. They have been installed on 21 buildings, only eleven-story buildings, with a total roof area of 24,583 m^2^, which represents 3.6% of the roof area of all prefabricated buildings.The presence of photovoltaic cells does not, however, hinder the potential of the buildings to establish green roofs, as previously indicated.

**Fig 6:**
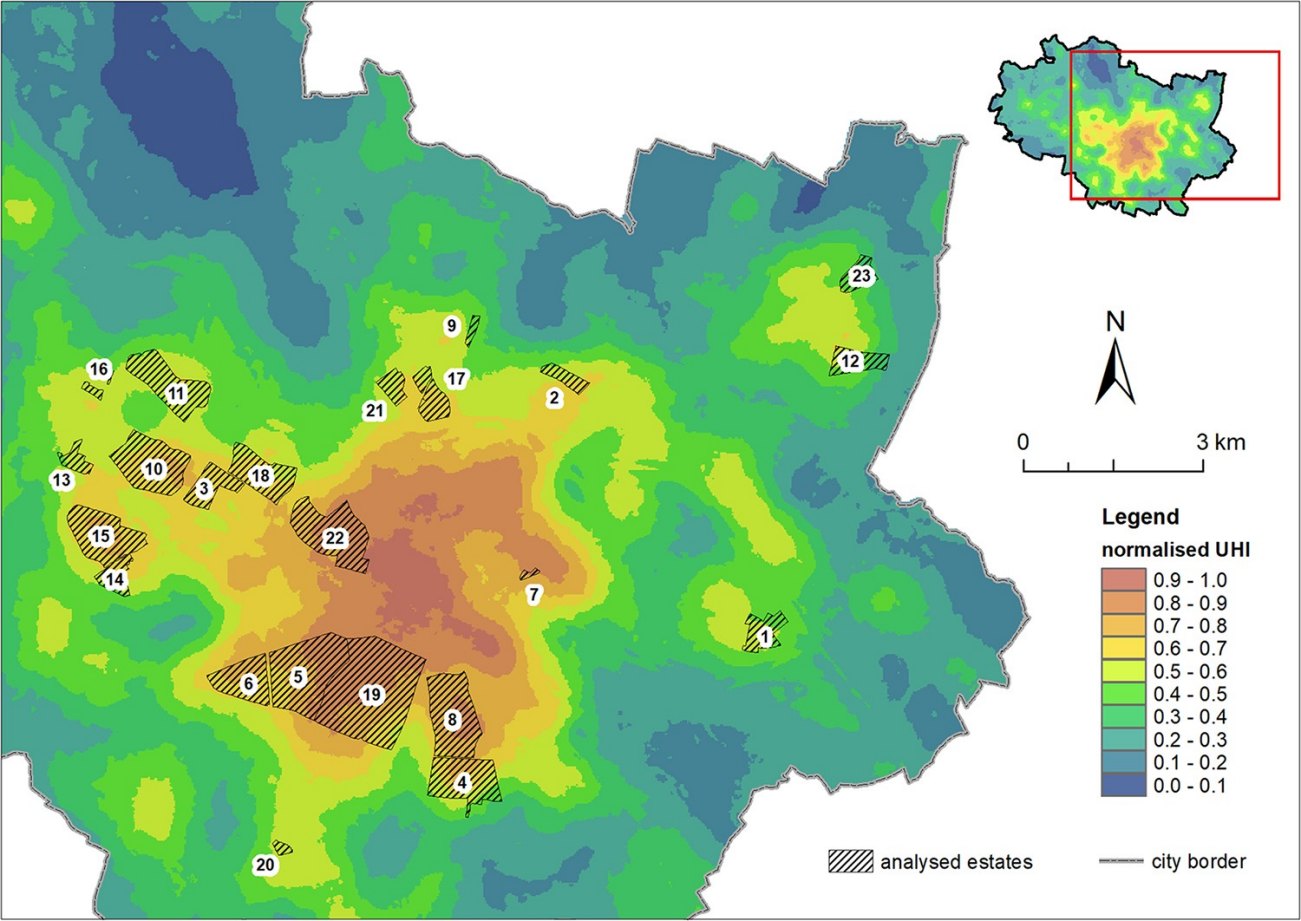
Distribution of the surveyed housing estates with respect to the normalised urban heat island in Wrocław (nUHI).

On the basis of the analysis of the distribution of the studied housing estates and the buildings located in them in relation to the nUHI (Fig 6), it has been shown that the largest number of buildings with a total roof area of 191,249m^2^ are located in the range of 0.6–0.7 nUHI; in the next zones the distribution of roof areas is as follows: in the range 0.5–0.6 nUHI the total roof area is 181,641m^2^, in the range 0.7–0.8 nUHI– 170,217m^2^, in the range of 0.8–0.9 nUHI– 120,140 m^2^. None of the buildings fall within the 0.9–1.0 nUHI range.

**Fig 7:**
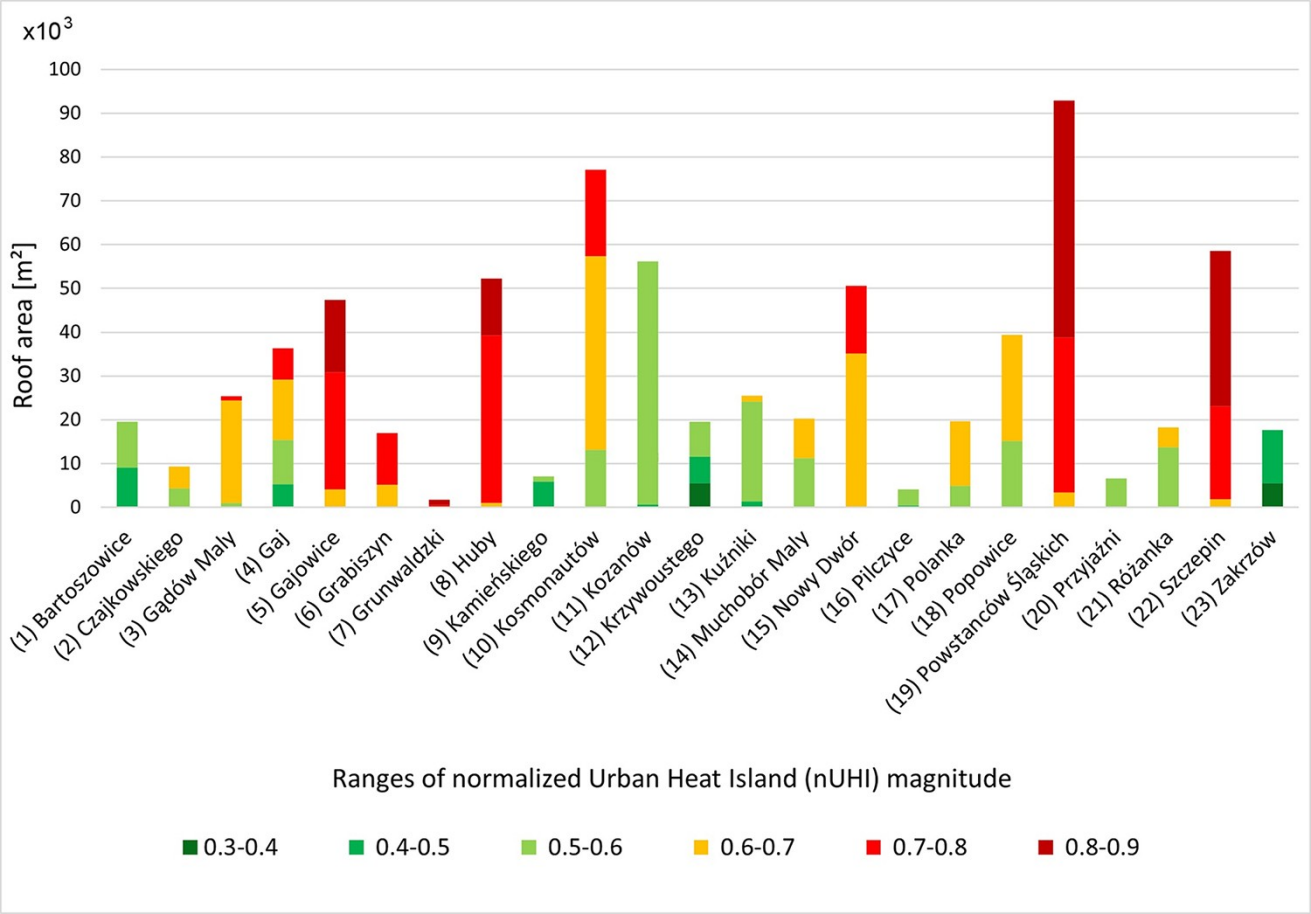
Roof area [m^2^] of prefabricated buildings located within areas of a given normalized Urban Heat Island (nUHI) magnitude. The numbering of the housing estates in accordance with Fig 2.

Most buildings in the>0.7 nUHI range class are located in the housing estates closest to the city centre: in the south–Powstańców Śl. (19), Gajowice (5) and Huby (8), in the west–Szczepin (21), in the north-east–pl. Grunwaldzki (7) (Fig 7).

**Fig 8:**
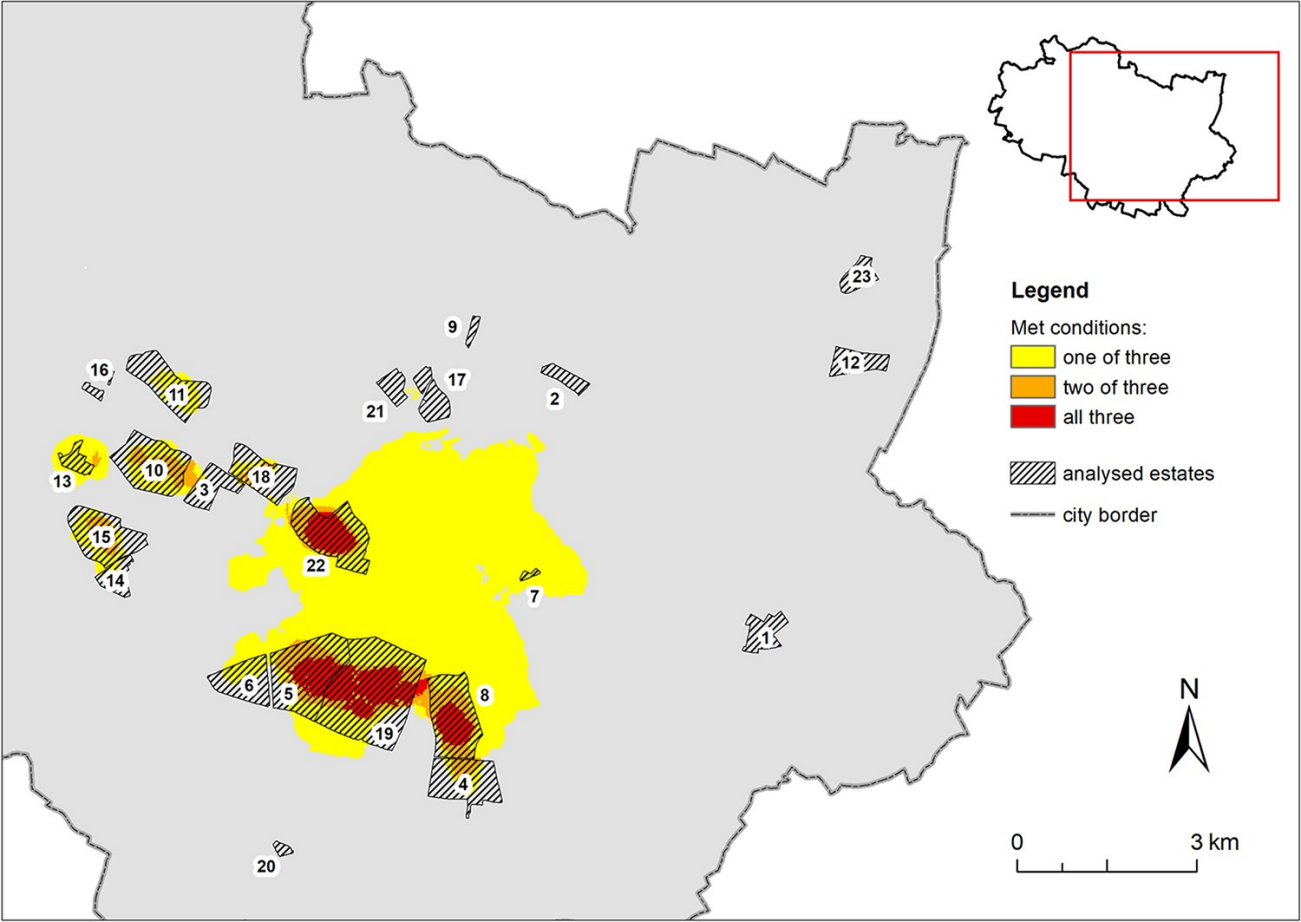
Delimitation of priority areas as meeting the criteria determined by Urban Heat Island of High Intensity (UHI HI), high Roof Surface Density (RSD) and high roof surface density of buildings with up to 5 floors (RSD_5H_).

In the subsequent steps of Stage II of the studies, RSD and RSD_5F_were determined by reclassifying the density maps. The obtained result areas were intersected with UHI HI (nUHI> 0.7) and areas that met one, two or all three criteria were identified (Fig 8).

**Fig 9:**
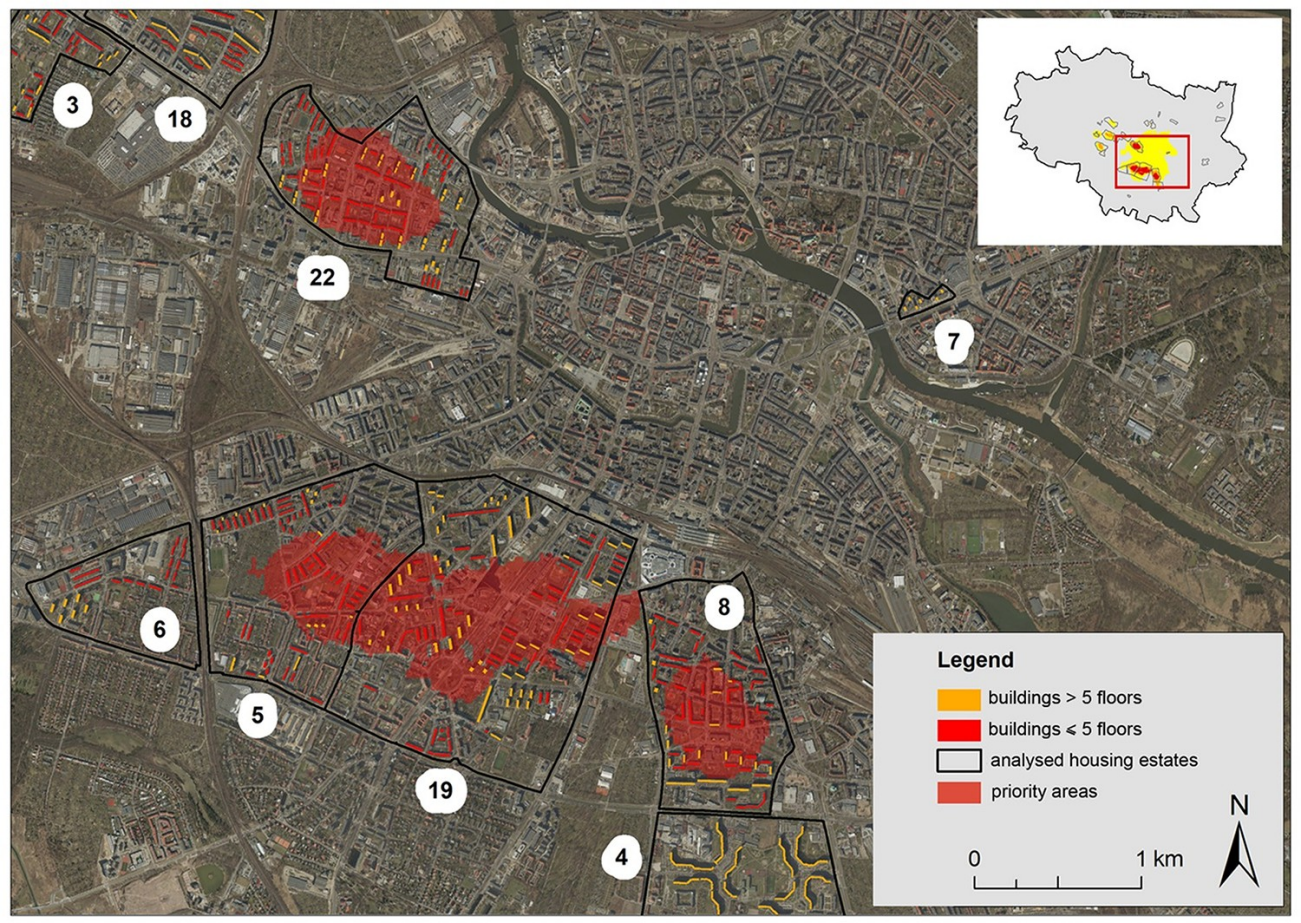
Priority areas where prefabricated building roof retrofits are envisaged first, presented on the orthophotomap 2020 (https://geoportal.wroclaw.pl/en/resources/).

Areas that fulfil all of the three criteria are priority zones where retrofitting of prefabricated buildings should be undertaken first (Fig 9).

They cover nearly 2 km^2^ and comprise a total of 480 buildings, with a total area of roofs 122749.1 m^2^, including 386 buildings of up to 5 floors with a roof area of 84368.6 m^2^ and 94 buildings of more than 5 floors with a roof area of 38380.5 m^2^. A summary of the number of buildings, their roof areas and the corresponding nUHI class ranges is presented in S1 Table.

In Stage III of the study, the structures of the buildings located in the priority areas were analysed to asses the RLC. All of the buildings in the priority areas were constructed in the Wroclaw Large Panel (WLP) system and their roofs were designed as prefabricated ventilated flat roofs. The system consists of prefabricated wall and floor panels, roofs and more complex elements including equipment and installations. The same reinforced concrete prefabricated elements were used for all buildings (lower and higher). They were delivered ready-made to the construction site, where they only had to be assembled, without the traditional division of work into so-called building shell and finishing work, which greatly accelerated construction. The prefabricated elements were made of vibrated gravel concrete with a strength of 200 kG/cm^2^, reinforced with steel bars (steel 34GS) as the load-bearing reinforcement and steel mesh (steel St0) as the distribution reinforcement.

**Fig 10:**
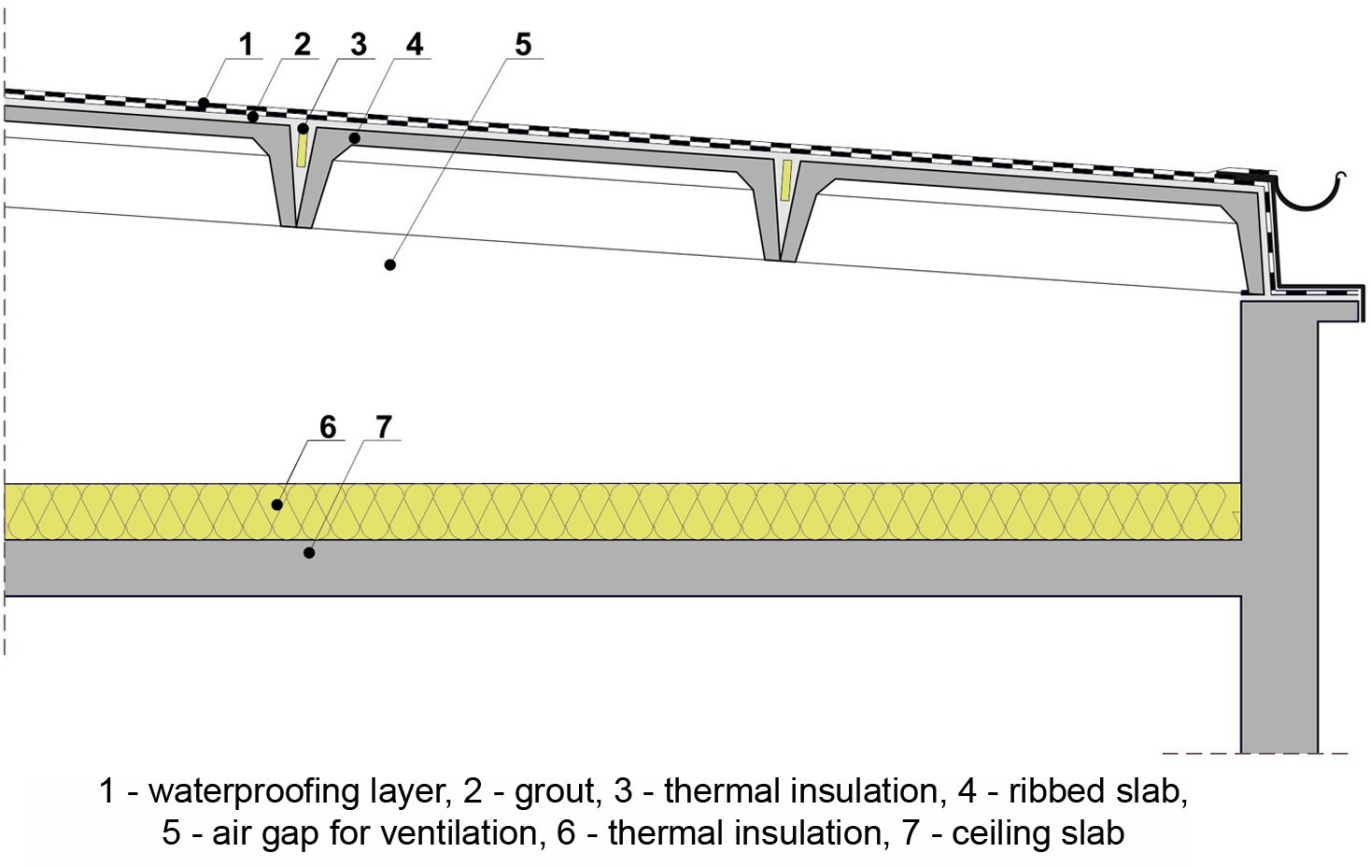
Roof cross-section of a typical building in the Wroclaw Large Panel (WLP) system.

The roof structure of large-panel buildings consists of prefabricated roof panels supported on prefabricated walls. Between the roof panels and the ceiling there is an air void, and thermal insulation is laid on the top of the roof. The roof panels are ribbed slabs with characteristic cross ribs and a rib along the perimeter. These slabs are 14 cm thick, 25 cm high, 120 cm or 150 cm wide and have two spans of 480 cm and 540 cm. The arrangement of the roof panels determines the RS, the angle of which in this case is 5°. When assembling the roof, the panels were covered with a cement layer and hydro insulation consisting of two layers of tar paper with adhesive (waterproofing layer) (Fig 10).

**Fig 11:**
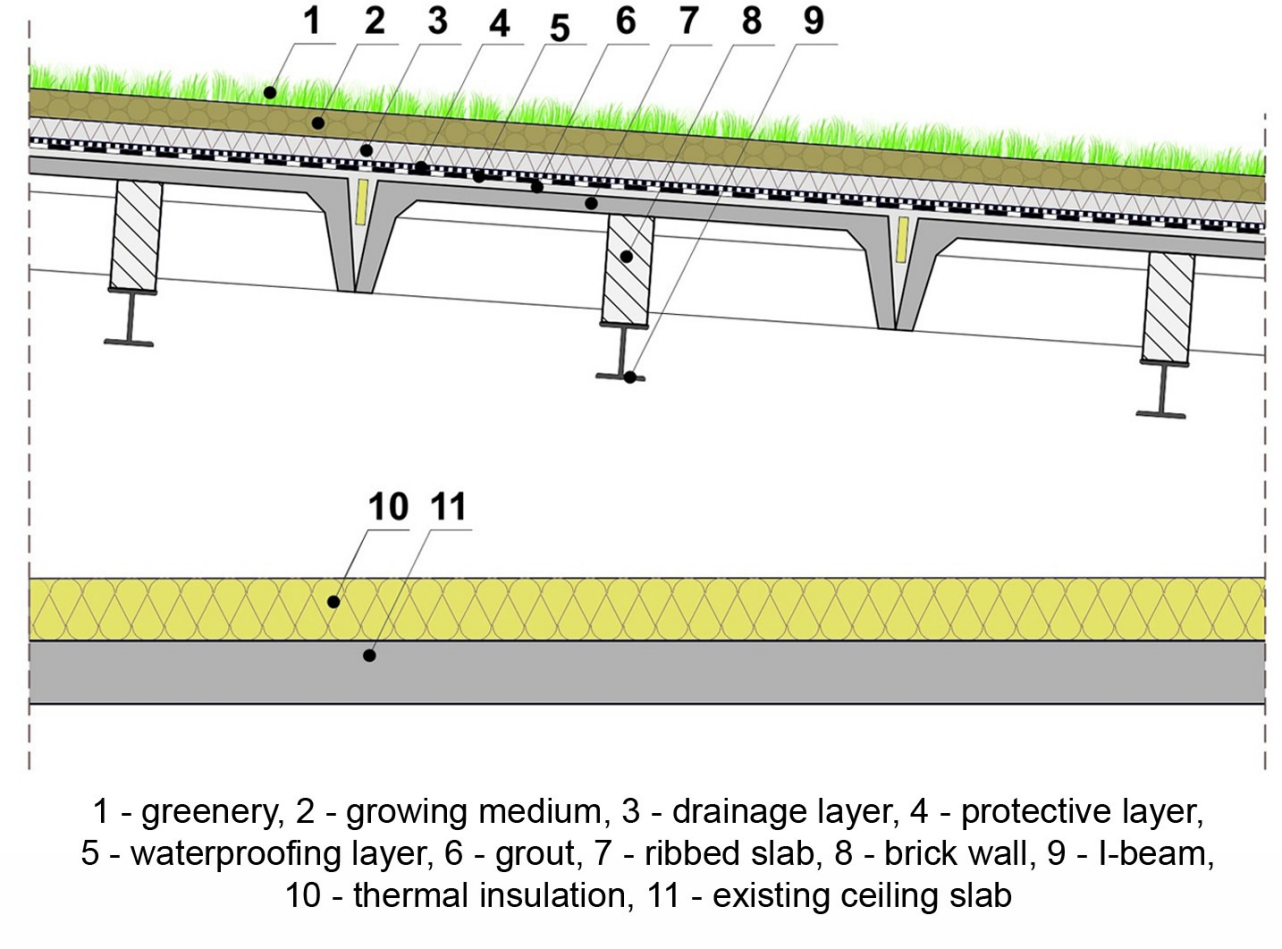
Extensive green roof on the existing roof structure–scenario 1.

The elements of the large-panel system, including ribbed slabs of the roof, were designed for the snow load zone II, i.e., for the roof load of 0.72 kN/m^2^ (using the standards in force during the period of the buildings construction [79, 88]. They were designed with a certain reserve since the buildings were and are located not in the 2nd but in the 1st zone of snow load. This reserve is 0.16 kN/m^2^ and the value of acceptable snow load is 0.56 kN/m^2^ (for roof load calculations according to snow loads standards [88, 89] see S2 and S3 Tables). The lack of available information on the load capacity of panelling makes it impossible to give a precise value of the additional load that the roofs could take in addition to the snow load. This information is not contained in the designs of buildings analysed under investigation, nor in the available subject literature. The ribbed slabs were not designed for high additional loads. It was not intended that the roofs would be developed and used for various purposes. The possible load above the own weight of similar slabs with a thickness of 15 cm, height of 30 cm, width of 149 cm and span of 5.97 m used in other large-panel systems designed in Poland is 1.42 kN/m^2^ [90]. Roofs with such larger slabs, apart from snow load and load from surface layers (cement– 0.21 kN/m^2^, waterproofing– 0.15 kN/m^2^), could carry a green roof load of up to 0.5 kN/m^2^, which would mean that only very light extensive green roofs could be introduced, provided that they are in good technical condition. Based on the above calculations, it can be concluded that with the panels as found on the roofs of the buildings under study, the loads resulting from the application of even very light green roofs would be exceeded. Therefore, as the first possible solution, it is proposed to build strain relief structures in the form of steel beams (steel I-beams) HE120A fixed in the roof space to prefabricated reinforced concrete walls and to fill the space between them and the roof ribbed panels with masonry construction (scenario 1) (Fig 11).

The reinforcement proposed in this way would allow for the establishment of extensive green roofs. The implementation of extensive green roofs would have to be preceded an on-site assessment of the technical condition for each roof, and in justified cases also by testing the strength of concrete ribbed slabs and their consequent reinforcement.

**Fig 12:**
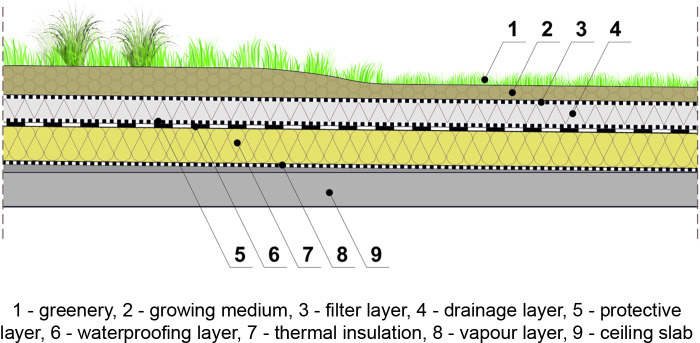
Semi-intensive green roof on the rebuilt roof structure–scenario 2.

The second possible solution (scenario 2) (Fig 12) is the reconstruction of the roofs by removing the present ribbed (pan) slabs and their supporting internal concrete partitions and erecting a green roof on the top floor slab of the last storey of the building.

The floor slab, according to the building plans and literature [79], is a solid slab 14 cm thick and can take an additional load of 3.64 kN/m^2^. Taking into account the snow load of 0.56 kN/m^2^, the load of new waterproofing layer, new thermal insulation layer, and equalizing layers (assuming the weight of selected materials available on the construction market), the load reserve is 2.31 kN/m^2^, which allows for the introduction of semi-intensive roofs. In view of the above, in scenario 2, we proposed a semi-intensive roof with a weight of 0.9 kN/m^2^. The stock of loads resulting from the use of this solutions is 1.41 kN/m^2^. Roof load calculations for the extensive green roof system are given in S4 Table, and for the semi-intensive roof system in S5 Table.

Simple intensive roofs are characterized by a vegetation layer that is at least 12 cm, 15 cm or 20 cm thick, depending on the planned vegetation. With regard to the planned vegetation, e.g., grass-herbaceous greening and wild perennial-shrub the greening would require a minimum substrate layer of 12 cm, wood-shrub-perennial greening would need a layer of min. 15 cm and wood-plant greening a min. 20 cm [83]. One of the possible semi-intensive roofs that could be used in scenario 2 could be the roof referred to as natural roof. The substrate used there is 6 cm thick, and on its fragments, it is built (formed) in the form of elevations up to 21 cm high, which allows for the introduction of a more varied selection of plants and creation of a habitat for a greater number of animal species [91].

The semi-intensive roofs proposed by the authors as a solution in Scenario 2 provide greater microclimatic and environmental benefits than the extensive roofs proposed in Scenario 1, although the construction of the former will require greater financial outlays resulting mainly from the necessary roof redevelopment. The final choice of the type of green roof to be used should, however, be subject to further study, including among others consideration of the opinions of local residents.

## Discussion

### Research procedure in the context of other approaches to the assessment of building rooftops for greening purposes

The procedure used in our study combines architectural, construction and climatic analyses, which distinguishes it from other approaches used in assessing the possibility of greening the roofs of existing buildings [40–54]. When analyzing buildings and their roofs and determining priority areas, various indicators were used, both those already known from other studies as well as completely new ones. The RAA assessment allowed us, like other scholars, to assess the external suitability of roofs for greening. However, the roofs of the analyzed buildings were not transformed into terraces, there are no stairs, HVAC plant and equipment and other elements that would divide their surface and make it difficult to introduce greenery, as demonstrated in related studies [41, 42, 50, 51, 82]. For this reason, we also did not define the smallest area for greening, which in turn was practiced in other studies, e.g. Grunwald defined the minimum specific area for greening as 10 m^2^ [51], Zhou– 100 m^2^ [50]. The next parameter–the number of floors (NF) is known from research on the assessment of the greening potential of buildings [40]. In our procedure, NF became the basis for classifying buildings into two types: up to 5 and above 5 floors, and at the same time for selecting a relatively homogeneous group of buildings whose roofs are low in relation to the ground level. In many studies on the relationship between the city climate and the urban form, especially with the use of Local Climate Zones (LCZs) [cf. 92, 93] the division into low-rise buildings (LRB), medium-rise buildings (MRB) and high-rise buildings (HRB) is used [e.g. 36, 37, 93]. We postulate the application of this universal three-level division in future studies involving different types of buildings, especially those for which no other classifications have been established.

To indicate the priority areas, we used three new indicators: location within the UHI of highest intensity (UHI HI), large roofs surface density (RSD) and large roof surface density of buildings with up to 5 floors (RSD_5H_). We assumed that buildings located in such designated areas should be selected for retrofit in the first place because of their greatest potential impact on UHI magnitude reduction. This assumption resulted from research proving that with the increase of the green roof surface, its microclimatic impact increases both on the scale of the neighbourhood [e.g. 30, 31] and the city [10, 26]. It was also important, as we mentioned before, that lower-lying green surfaces have a greater effect on cooling at pedestrian level and provide an opportunity for local changes in heat balance within the urban canopy layer [23, 26–31, 86].

Checking our assumptions and examining the cooling effect of green roofs in priority areas would require in situ research and/or simulation using, for example, the ENVI-met software.

When selecting buildings eligible for greening, we did not take into account the urban pattern, which also has an impact on the cooling effect [e.g. 27, 28, 30, 86]. The analyzed housing estates are characterized by a variety of plans and building layouts. This diversity results from the designers’ striving to obtain an individual character of the space, despite the typification of buildings [77]. The influence of the configuration of buildings and weather conditions on the ambient temperature in a specific location could, however, be considered in the next stage of the research.

The layout and geometry of buildings can also affect the shading or insolation of roof slopes. Permanent shading is considered a major constraint on the introduction of greenery [e.g. 48, 82], or, as Castleton et al. [46] point out, drawing on the findings of Getter et al. [94], requires a special selection of plant species tolerant of such habitat conditions, and therefore is of importance to the adoption of the final solution. In the case of prefabricated housing estates, the conditions for good sunlight in dwellings were strictly observed [95] and the distances between buildings were kept accordingly, so the criterion of roof shading did not have to be taken into account in the research. However, when housing estates become densified with new buildings, the insolation and shading of roof surfaces will change, and this aspect would need to be investigated.

The third stage of the research, involving the structural analysis of buildings located in the priority areas, including the assessment of RLC, was of particular importance for the presentation the roof greening scenarios and, to the greatest extent, determined the implementation character of the findings. We confirmed the ability of reinforced concrete structures to bear additional loads [45–47] and indicated the limitations in the installation of green roofs resulting from the current construction of prefabricated ventilated roofs (“cool roofs”). Such a level of research detail is only possible with regard to selected types of buildings or areas with predominantly homogeneous development. In the case of investigations assessing the potential of buildings to establish green roofs performed for entire cities e.g., Grunwald et al. [51] for Braunschweig, Velázquez et al. [54] for Madrid, Silva et al. [40] for Lisbon, Hong et al. [52] for Shenzhen the inclusion of RLC is hampered by the scale of the area and/or the lack of building stock data that could be used in such studies [51]. The omission of the RLC in the studies makes it possible to assume that the area to be greened determined on their basis is overestimated, and will be reduced once the construction aspects are considered.

In many procedures of assessing the suitability of existing buildings to introduce green roofs the parameter taken into account is the RS [e.g. 40, 51, 52, 54]. It is assumed, i.a. that roofs with RS up to 10° do not require anti-slip solutions, unlike roofs with higher slopes [83]. In our study, focused on one type of development–prefabricated apartment blocks–it was known in advance that the analyzed roofs were flat roofs, and their exact slope, was verified when analyzing the structure, based on construction designs. In the procedures for assessing the development potential for green roofs, which covered entire cities and many types of buildings, RS analyzes were performed in the first place on the basis of satellite images [40, 52] or high-resolution numerical terrain models [51, 54]. This approach is fully justified when taking into account many types of buildings, but it could be omitted in our study.

### The roof retrofit scenarios and their future implementation

The proposed scenarios for green-roof retrofitting show possible structural solutions and determine the type of green roof to be applied. Each scenario has both its limitations and strengths. The first of them, involving the reinforcement of the roof structure, will be easier to implement, but its impact on UHI reduction will most likely be smaller than in the second scenario, which assumes the use of semi-intensive greening. However, it would be possible to check the impact of individual solutions, as already mentioned, in the next stage of research–using the ENVI-met software or/and experimental studies with green roof models, as e.g. performed by Zhang et al. [24]. The final solution should consider not only the selection of plants, but also irrigation, as its lack, may lead to the opposite effect–an increase in temperature [24].

The final choice of a roof retrofit scenario should be preceded by research on the needs and preferences of urbanites. Different types of well-known methods of participatory planning [96] would provide the basis for designers to realise a vision in line with local expectations [see 97 for more]; they could also help raise public awareness of the importance of green roofs, as they are still a relatively new solution in Poland.

In addition to the scenarios presented in the results for converting roofs to green roofs, there is also the option of using a cool roof–covering the roofing paper layer or concrete with a material that does not absorb sunlight. This solution included alongside green roofs in the UHI mitigation strategies [10, 98, 99] would not require reinforcement of the structure of current roofs or their rebuilding, so it would be the simplest option with the lowest cost, but it would lack many of the other benefits that green roofs can provide in terms of rainwater retention and slowing down its runoff, biodiversity, aesthetics, among others [1–6].

When it comes to the possibility of implementing green roofs on the examined buildings, an appropriate city policy could be of great support in this regard, while its lack could be a significant barrier [7, 100]. It seems that in the case of Wrocław financial programs would be very important (e.g., as part of the city’s adaptation to climate change and minimizing its effects). Currently, the costs of all modernization works are borne by the residents of the buildings–members of housing cooperatives or communities.

### Limitations

In the process of designating priority areas, we used data on the spatial structure of the UHI from the years 2001–2002, which from a methodological point of view is not very important, but the final recommendation for local authorities should be based on updated figures on the UHI range and magnitude. Not all cities hold data on the UHI, and the preparation of such spatially continuous information is rather complicated and time-consuming (especially field measurements and the subsequent spatial interpolation). On the other hand, it is relatively straightforward to obtain land surface temperature data from satellite imagery, which of course are considered to be a reliable indicator of the UHI, but the conversion of land surface temperature into the classical UHI is not readily apparent [cf. 101]. Therefore, high-resolution physical modelling is becoming a real alternative to measurement and remote sensing data. Many studies relating to urban climate are based on microclimate modelling using specific software, e.g., ENVI-met [e.g. 28, 30, 31], or, for larger areas, mesoscale climate modelling using e.g. Weather Research and Forecasting (WRF) model [102, 103].

It should also be noted that research on prefabricated buildings may be limited by the lack of data on the load-bearing capacity of individual prefabricated elements used for its construction, including the load-bearing capacity of roof panels. We have not been able to find data on the possible additional load of the roof ribbed panels above of their own weight. These data were neither available in the literature on prefabricated construction from the second half of the 20th century [79, 87] nor in the design of buildings. Therefore, we finally estimated the load capacity of the roof panels on the basis of data concerning similar ribbed reinforced concrete panels [90]. Such limitations may appear likely when analyzing subsequent prefabricated buildings. Despite the similarities in their structure, there were differences in the size and reinforcement of the elements (walls, ceilings), and thus their load-bearing capacity. In Poland alone, there were 8 large-panel prefabricated systems, the so-called central (used throughout the country) with their 8 varieties and 6 regional systems, used only in certain parts of the country [87].

### Prospects for further research

The presented research, as mentioned previously, could be extended with simulations of temperature changes in priority areas using computer modelling.

An interesting issue would also be to examine the thermal insulation of the roof before and after the introduction of greening. It is highly probable that it has low thermal insulation, both because of the time which has passed since the buildings were constructed (35–50 years) and the wear and tear of the material used for insulation (mineral wool). The conversion of the roof into a green roof would therefore be further justified by the improvements in thermal insulation. Thermal retrofits are being forced by the new version of the EU Energy Performance of Buildings Directive [104]; it requires EU Member States to establish a long-term strategy to facilitate the cost-effective conversion of existing buildings into nearly zero-energy buildings. This means that all newly designed buildings and buildings undergoing thermal retrofitting are to be designed with near-zero energy consumption in mind. According to the Buildings Performance Institute Europe, up to 97% of buildings in the EU need to be upgraded [105]. At the same time, it is the older buildings, built to completely different thermal insulation standards than the current ones, that benefit most from the installation of green roofs [46], moreover, it is believed that green roofs have almost no effect on the annual energy consumption of new buildings built in accordance with current European regulations requiring high levels of insulation [46].

In the future, research into the greening potential of the roofs of the studied buildings could take into account other prefabricated apartment blocks (built in other large-panel systems or other prefabricated technologies) and additional environmental criteria. This would first verify the load-bearing capacity of other roofs and identify further areas where the application of green roofs would provide needed ecosystem services, taking into account both synergies and trade-offs between these services [cf. 53].

It seems also that the development of green roof technology should go in the direction of seeking the lightest possible structural solutions, which would make it possible to introduce greenery on roof structures with low bearing capacity, without the need for their more expensive reconstruction.

## Conclusion

The presented study provides knowledge on how to green the roofs of prefabricated apartment blocks and proposes how to select these buildings in order to alleviate UHI. The study used a three-stage research procedure consisting of architectural, construction and climatic analyzes. In addition to the known and used indicators, such as RAA and RCL, allowing to determine the possibility of installing green roofs, new ones were introduced–UHI HI, RSD and RSD_5F_. They made it possible to identify priority areas, i.e., those in which roof retrofitting should be undertaken first due to UHI magnitude.

The procedure may be applied in any other city in the world provided that data on the buildings and structure of the UHI zone are available. The adopted values of UHI HI, RSD and RSD_5H_ indicators can be modified depending on the specificity of buildings and the size of UHI, and the procedure itself can be extended with new parameters justified by local conditions.

It has been shown that the priority areas are located in the city centre and cover the area of 2 km^2^. They include 480 buildings with a total roof area of 122749.1 m^2^. It has been shown that the construction of prefabricated roofs creates certain limitations in greening, despite their external suitability. The proposed transformation scenarios assume: (a) strengthening the structure of existing ventilated flat roofs (“cool roofs”) and introduction of extensive greening, (b) conversion of ventilated flat roofs to unventilated ones and introduction of semi-intensive greening.

The presented study fills the gap in the research on the modernization of prefabricated housing buildings with the use of green roofs. The obtained results have a practical dimension and may constitute the basis for design works, consultations with residents and building managers, and administrative activities supporting the implementation of green roof systems on existing buildings.

## Supporting information

S1 Table Summary of the results from the 1st and 2nd stage. (TIF)

S2 Table Roof surface loads assumed in the original design of buildings in the Wrocław Large Panel system (WLP) according to PN-80/B-02010 Loads in static calculations–Snow loads applicable during the construction of buildings. (TIF)

S3 Table Roof surface load for the building in the Wroclaw Large Panel system (WLP) according to the currently applicable PN-EN 1991-1-3:2005 Eurocode 1: Actions on structures–Part 1–3: General actions–Snow loads. (TIF)

S4 Table Roof surface load for the building in the Wrocław Large Panel system (WLP) according to the currently applicable PN-EN 1991-1-3:2005 Eurocode 1: Actions on structures–Part 1–3: General actions–Snow loads with the use of an extensive green roof. (TIF)

S5 Table Roof surface load for the building in the Wrocław Large Panel system (WLP) according to the currently applicable PN-EN 1991-1-3:2005 Eurocode 1: Actions on structures–Part 1–3: General actions–Snow loads with the use of a semi-intensive green roof. (TIF)
