# Supplementary figures and images for: Procedure for the selection and evaluation of prefabricated housing buildings for the implementation of green roofs in the context of Urban Heat Island mitigation. The example of Wrocław, Poland
Source: PLoS One. 2021 Oct 14;16(10):e0258641. doi: 10.1371/journal.pone.0258641 (PMC8516204; doi:10.1371/journal.pone.0258641)

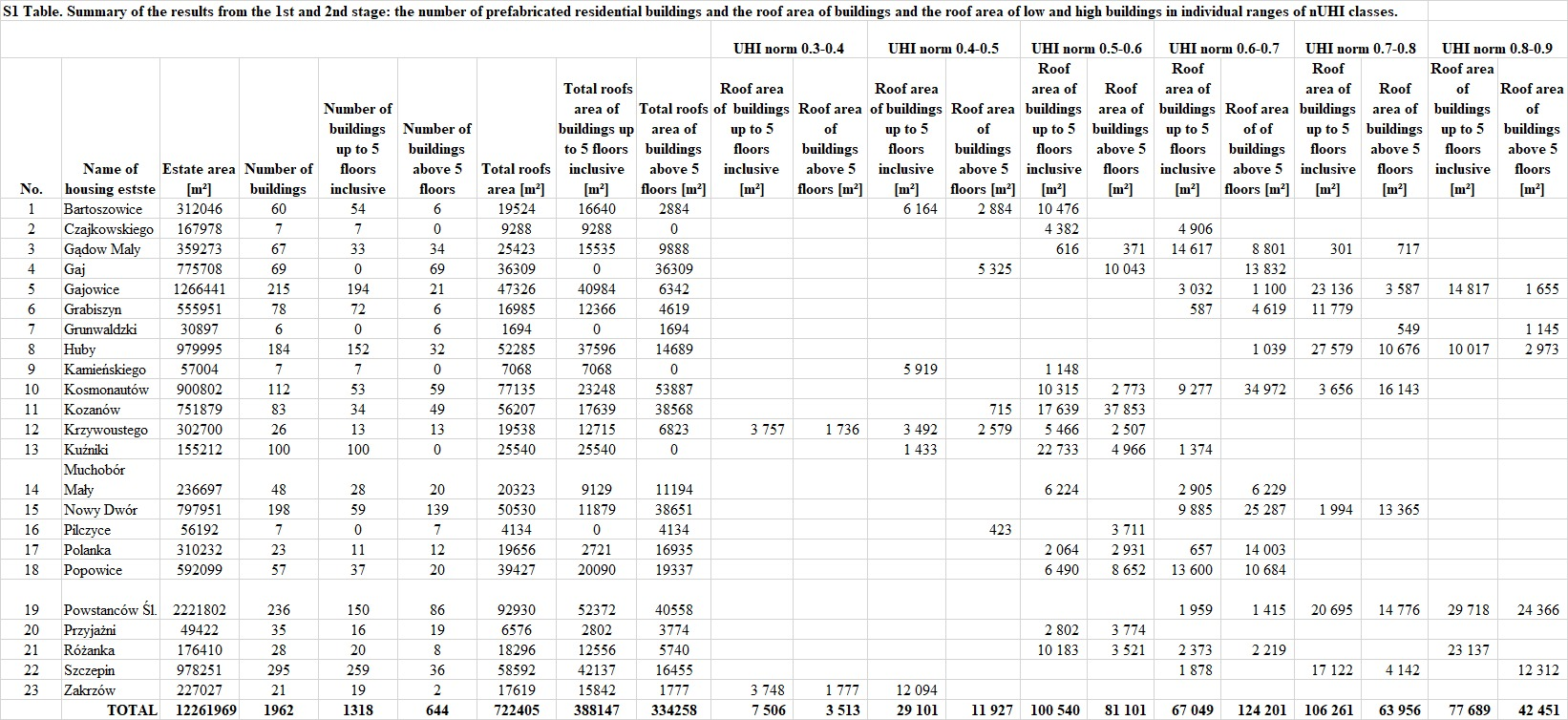

Supplement: S1 Table — (TIF) [file pone.0258641.s001.tif]

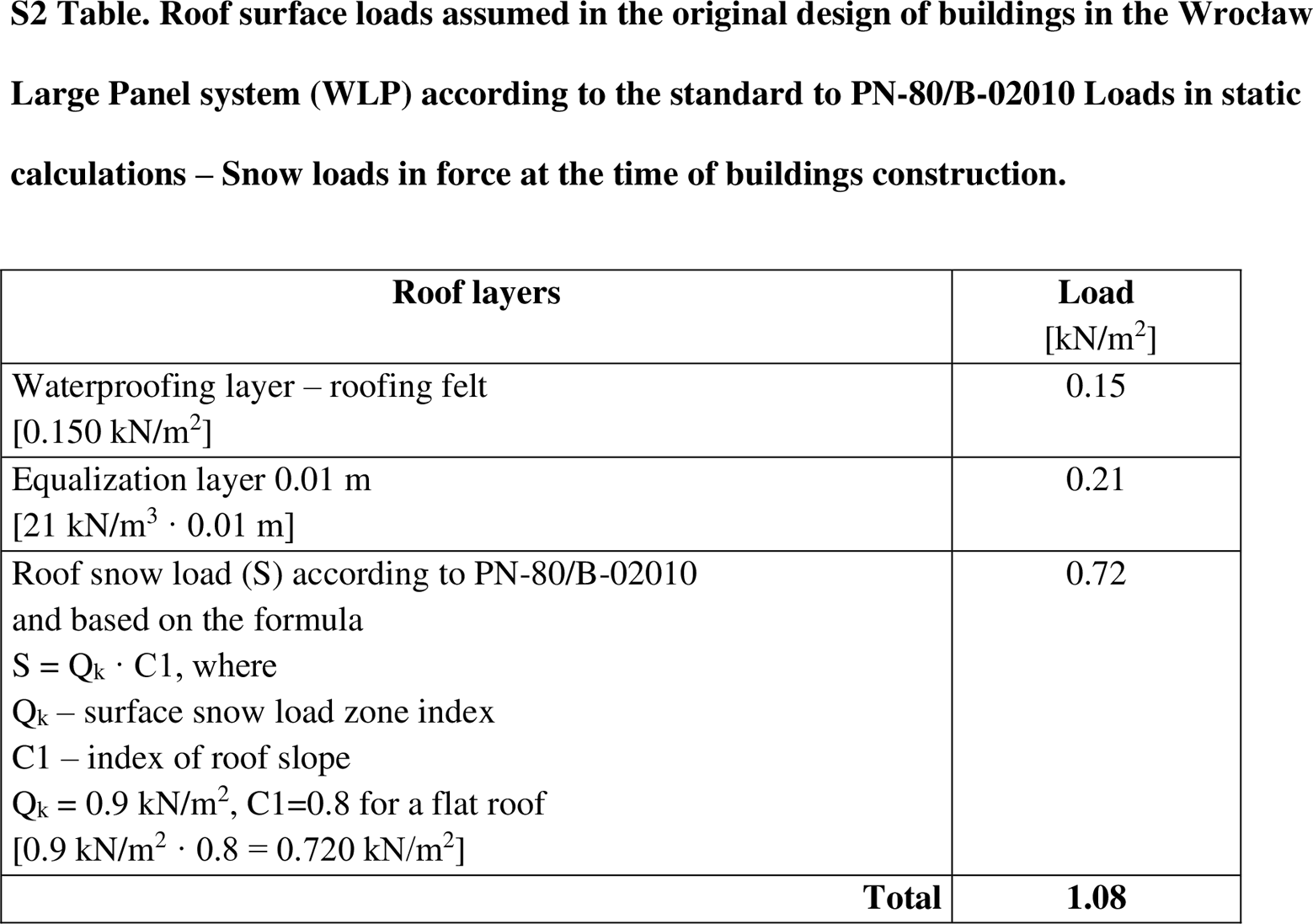

Supplement: S2 Table — (TIF) [file pone.0258641.s002.tif]

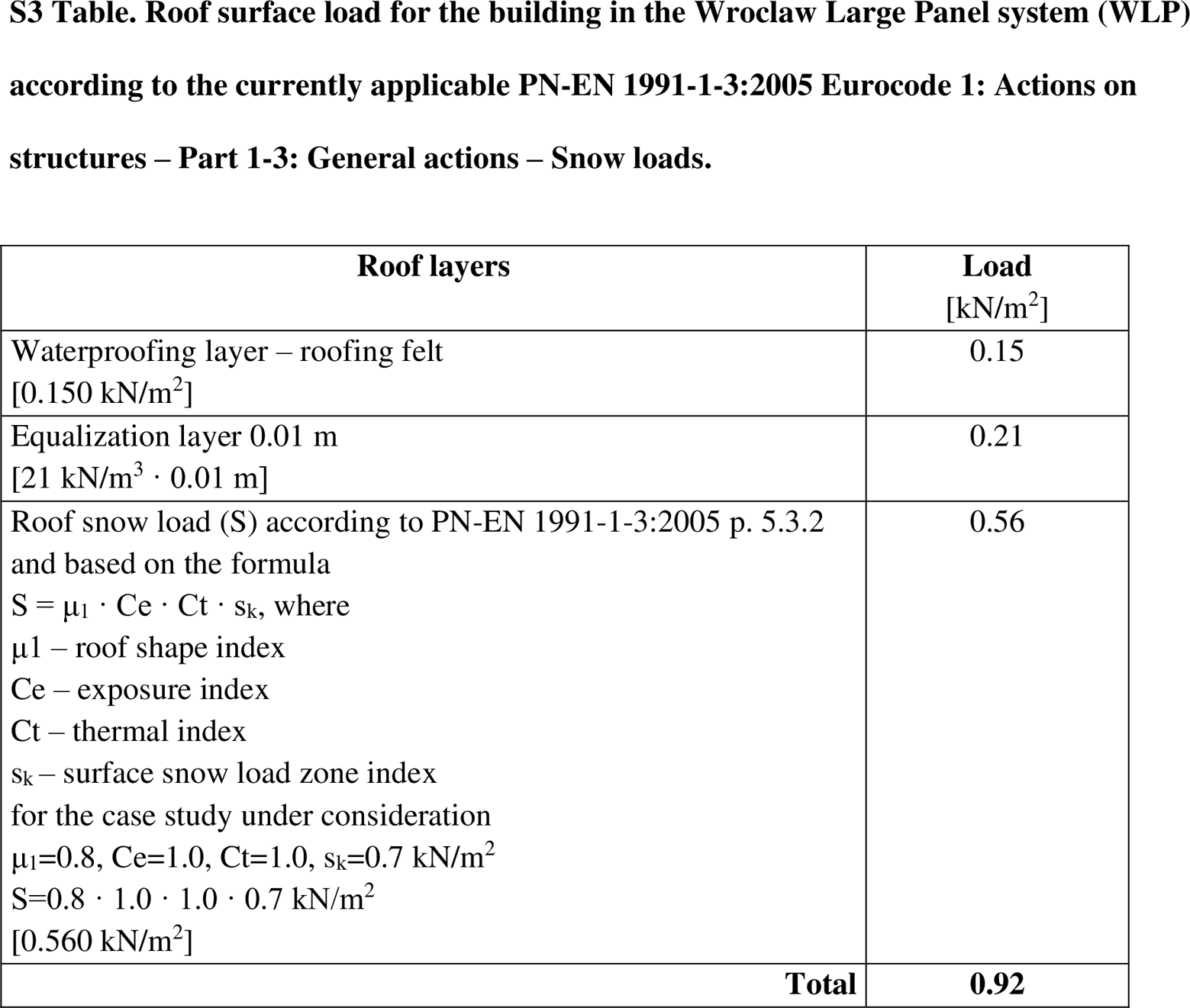

Supplement: S3 Table — (TIF) [file pone.0258641.s003.tif]

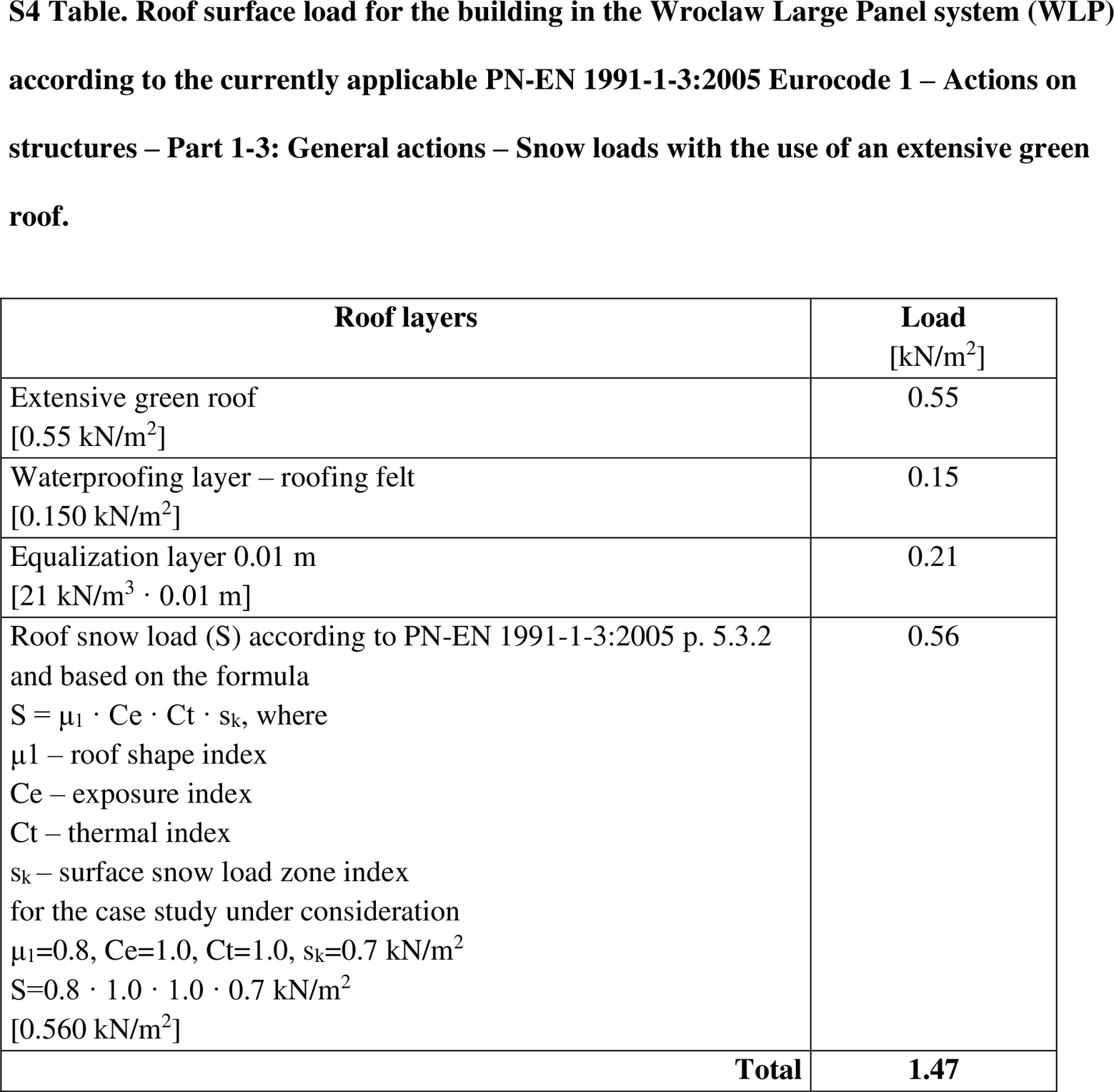

Supplement: S4 Table — (TIF) [file pone.0258641.s004.tif]

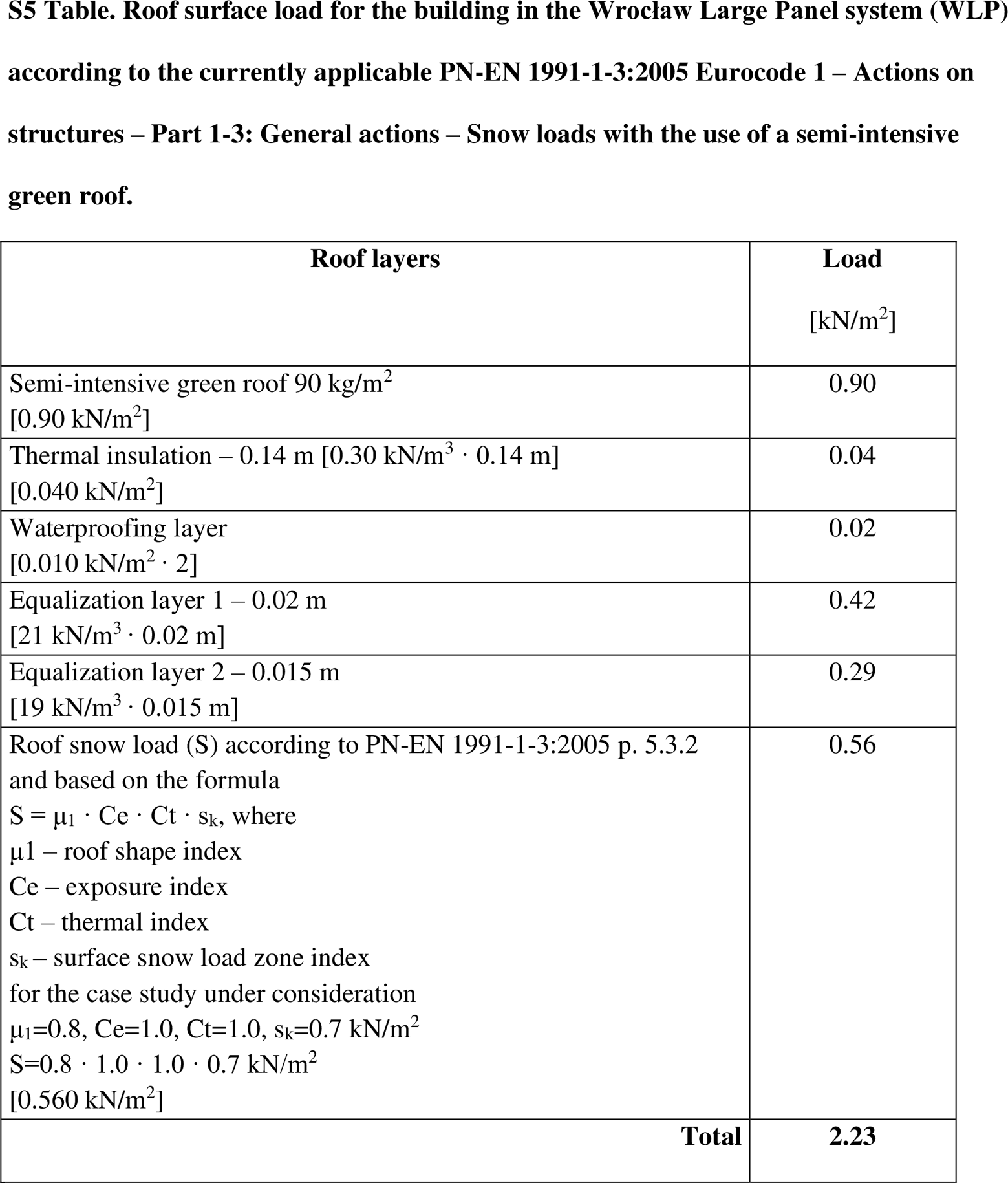

Supplement: S5 Table — (TIF) [file pone.0258641.s005.tif]
